# Intrauterine G-CSF Administration in Recurrent Implantation Failure (RIF): An Rct

**DOI:** 10.1038/s41598-020-61955-7

**Published:** 2020-03-20

**Authors:** Ziya Kalem, Muberra Namli Kalem, Batuhan Bakirarar, Erkin Kent, Antonios Makrigiannakis, Timur Gurgan

**Affiliations:** 1 Istinye University Liv Hospital Bahcesehir, Department of IVF, Istanbul, Turkey; 2 Bahcesehir University, Department of Obstetrics and Gynecology, Istanbul, Turkey; 3 Ankara University, Department of Biostatistics, Ankara, Turkey; 4 Gürgan Clinic IVF and Women Health Center, Department of Embryology, Ankara, Turkey; 5 Crete University, Department of Obstetrics and Gynecology, Crete, Greece

**Keywords:** Biological therapy, Randomized controlled trials

## Abstract

This study investigates the effects of intrauterine G-CSF on endometrial thickness, clinical pregnancy rate and live birth rate in a recurrent implantation failure (RIF) group with normal endometrium. This study was designed as a prospective randomized controlled trial with the involvement of 157 RIF group pati; ents. The RIF group was formed on the basis of the RIF criteria: “The failure to achieve a clinical pregnancy after the transfer of at least four good-quality embryos in a minimum of three fresh or frozen cycles to a woman under the age of 40 years. The study sample included 82 patients in the G-CSF group who received G-CSF once a day on hCG. The procedure was performed by administering 30 mIU of Leucostim®(Filgrastim [G-CSF] 30 mIU/mL; DEM Medical, Dong-A; South Korea) through slow infusion into the endometrial cavity using a soft embryo transfer catheter. Normal saline of 1 mL was infused into the endometrial cavity in the same way in 75 patients in the control group. The standard ICSI procedure was used for all patients, and fresh cycle embryos were transferred on the third or fifth day. No statistically significant difference was identified in clinical pregnancy rates, miscarriage rates and live birth rates between the G-CSF group and the control group (p = 0.112, p = 0.171, p = 0.644, respectively), and no difference was observed between the two groups regarding endometrial thickness (p = 0.965). The intervention of administration G-CSF into the uterine cavity in RIF patients with normal endometrium, did not alter the endometrial thickness, clinical pregnancy rates, or live birth rates.

## Introduction

Recurrent implantation failure (RIF) refers to a situation in which implantation has repeatedly failed to reach a stage recognizable by pelvic ultrasonography^1^. Coughlan *et al*. proposed that RIF is defined as the failure to achieve a clinical pregnancy after the transfer of at least four good-quality embryos in a minimum of three fresh or frozen cycles to a woman under the age of 40 years^2^. However, internationally agreed consensus on the definition has yet to be reached. Although the two leading actors in the implantation are the endometrium and the embryo, there are some other influential factors, such as oocyte and sperm parameters, parental chromosome structure, anatomic structure, immunologic factors, thrombophilic conditions and lifestyle, which means that a multidisciplinary approach is required for the management of RIF.

Granulocyte colony-stimulating factor (G-CSF) is a hematopoietic‐specific cytokine produced by bone marrow cells, stromal cells, fibroblasts, and macrophages^3^, although several nonhematopoietic cell types, such as endothelial cells, placental cells, trophoblasts and granulosa‐lutein cells, also express G‐CSF receptors^4,5^. G-CSF helps mainly in the mobilization, migration and differentiation of stem cells^6^, while also facilitates endometrial regeneration by promoting angiogenesis and decreasing cell death by reducing apoptotic activity^7^. G-CSF plays a role in embryo implantation and the continuation of pregnancy by temporarily suppressing immune response through its effects on lymphocytes, macrophages and T helper-2 cells^8^. Thus, G-CSF supplementation has been considered as a promising innovative therapy in reproductive medicine. G-CSF has also been reported to play a role in embryonic development, implantation and trophoblastic growth when administered systemically^9^ and have beneficial effects on endometrial remodeling and receptivity in intra-uterine local administration^10^.

Clinical trials that suggest that G-CSF administration may improve the success of the assisted reproductive techniques (ART) have been performed primarily as thin endometrium studies^11,12^. In recent years, studies into recurrent pregnancy loss and recurrent implantation failure have also reported successful outcomes^13,14^. In addition to studies that showed that G-CSF administration provides endometrium expansion, particularly in the thin endometrium, and increases implantation and pregnancy rates, there are also studies that found out that G-CSF administration does not change endometrium and pregnancy rates, although it is seen to increase the endometrial thickness^11,12,15–17^. The relationship between RIF and endometrium, G-CSF administration and endometrium has been studied extensively, although there have not been enough randomized controlled trials on G-CSF administration in the RIF group with normal endometrium. To contribute to the literature, in this study, we investigated the effect of intrauterine G-CSF administration on endometrial thickness, implantation rates and live birth rates in RIF group patients with normal endometrium.

## Materials and Methods

This study is designed as a prospective randomized controlled trial with 157 patients diagnosed with RIF without endometrial pathology and was carried out in a private infertility center between March 2016 and December 2017. The recurrent implantation failure group was formed on the basis of the RIF criteria defined by Coughlan *et al*. in 2014, being “the failure to achieve a clinical pregnancy after the transfer of at least four good-quality embryos in a minimum of three fresh or frozen cycles to a woman under the age of 40 years^2^.

Women under the age of 40 who met the RIF definition and patients whose follicle-stimulating hormone (FSH) levels were <15 IU/mL were included in this study. Patients with autoimmune diseases, patients with congenital uterine anomalies, patients with Asherman’s syndrome, patients with uterine cavity distorted by myoma or endometrial polyps, patients with confirmed endometriosis or endometrioma, and patients for whom G-CSF was contraindicated (active infections, kidney disease, sickle cell anemia, malignancies, chronic neutropenia) were excluded from this study. The patients with an endometrial thickness less than 7 mm (measured in the pretreatment midcycle) were accepted as thin endometrium and didn’t included this study.

This study was approved by the Ethics Committee of Turgut Ozal University and was conducted in accordance with the principles of the Declaration of Helsinki. To calculate the sample size, the percentages of clinical pregnancy rates were taken as 37.5% and 14.3% for the groups that received G-CSF and those that did not receive G-CSF respectively^17^ and the significance level was considered to be 0.05, and samples were measured using the Chi-Square test with a 0.80 power; as a result, findings showed that 74 samples per group and 148 samples in total were sufficient for this study.

**Figure 1:**
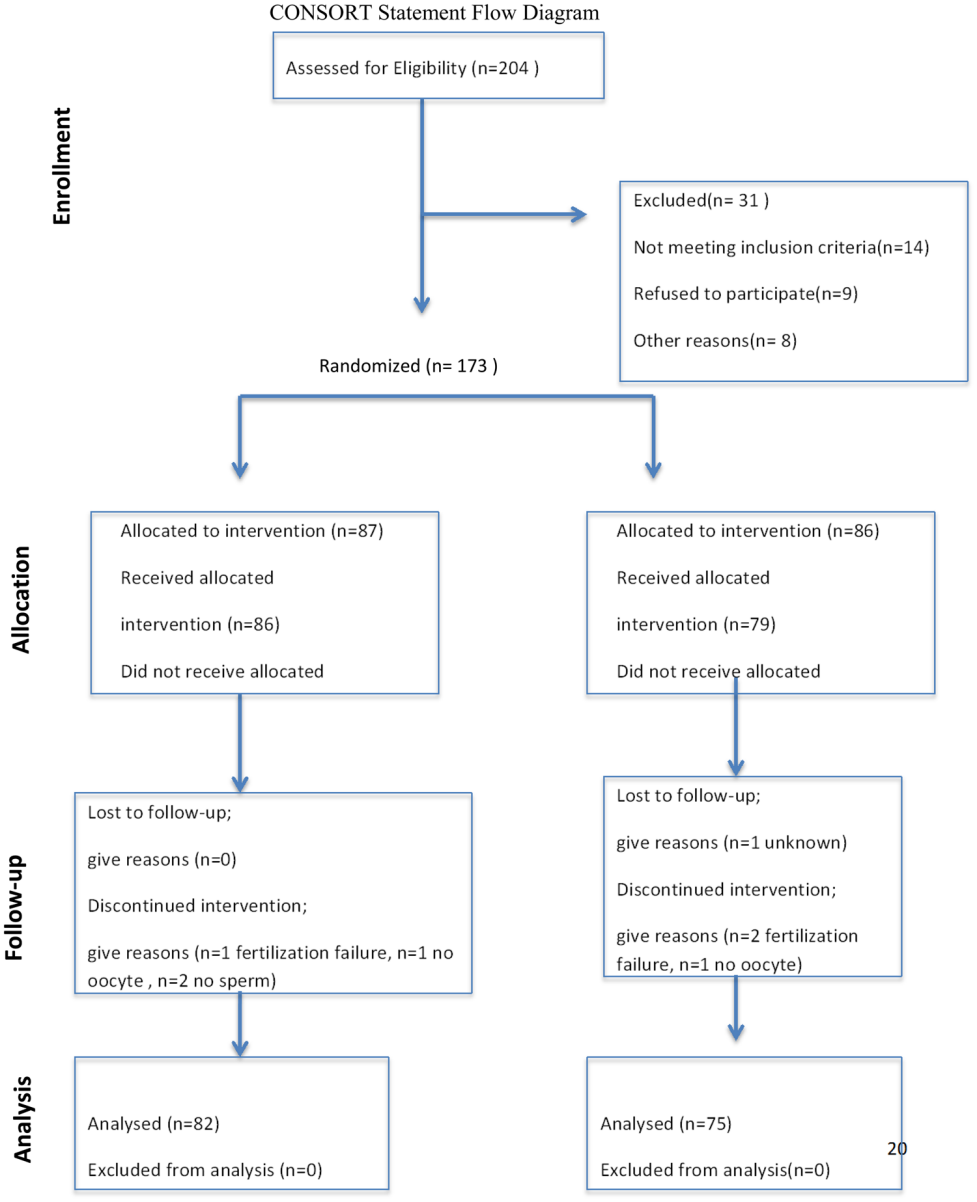
Consort Statement Flow Diagram that represents the participants flow in the trial.

For this study, 204 participants were evaluated, and 173 suitable participants were informed in detail about this study. Written informed consent was obtained, and the patients were then randomized using a computer-generated random number sequence (1:1 simple randomization). The patients were blind to which group they were assigned to, the physicians were not. The patients were subjected to randomization only once during this study and were not included in the study for the second time. This study was continued with a total of 157 patients, 82 of whom were in the G-CSF group, and 75 were in the control group. A CONSORT diagram that shows the status of the participants during this study can be found in Fig. 1.

All of the patients included in this study were followed by through two cycles. In the pretreatment cycle, age, BMI, infertility duration, FSH and AMH levels, number of antral follicles, sperm count and endometrial thicknesses in the periovulatory period were recorded, and the patients were randomized. In the intracytoplasmic sperm injection (ICSI) cycle, patients in the G-CSF group received G-CSF once a day on hCG day, before hCG injection. The procedure involved the administration of 30 mIU of Leucostim (Filgrastim [G-CSF] 30mIU/mL; DEM Medical, Dong-A; South Korea) through slow infusion into the endometrial cavity using a soft embryo transfer catheter. Normal saline of 1 mL was infused into the endometrial cavity of patients in the control group in the same way as the study group. A standard ICSI procedure was used for all patients, and fresh cycle embryos were transferred on the third or fifth day. The ICSI cycle parameters of all the patients and pregnancy outcomes after the ICSI cycle were recorded.

Ovarian stimulation protocols and luteal support: In this study, gonadotropin-releasing hormone (GnRH) agonist and GnRH antagonist protocols were administered to patients for ovarian stimulation.In the agonist protocol, GnRH agonist 0.5 mg/day leuprolide acetate (Lucrin Daily; Abbott, Istanbul, Turkey) was started in the luteal phase of the previous cycle and recLH treatments were added in addition to recFSH, HMG or recFSH on the second and third days of the cycle. Drugs used in these treatments included recombinant human follicle stimulated hormone (r-hFSH) follitropin-α (Gonal-F®; Serono, Geneva, Switzerland) or follitropin-β (Puregon®; Organon, Oss, the Netherlands), human menopausal gonadotropin HMG) (Menogon®; Ferring, Istanbul, Turkey) or highly purified human menopausal gonadotropin (HP-HMG) (Menopur®; Ferring, Istanbul, Turkey) and recLH (Luveris; Merck Serono, Istanbul, Turkey). In the antagonist protocol, when the gonadotropin was started on second and third days and the dominant follicle was 14 mm, cetrorelix (Cetrotide®; Merck Serono, Turkey) was added to the treatment as a GnRH antagonist. In both protocols, the development of the follicles was followed by transvaginal USG and E2. When the dominant follicle measurement was 19 mm or more or at least three follicles were 17 mm or more, gonadotropin administration was terminated and ovulation was induced. Ovulation was induced with 250 µg of human recombinant hCG (Ovitrelle®, Merck Serono, Turkey). Oocyte retrieval was performed during transvaginal USG process at between the 36th and 40th hour after the ovulation induction, followed by ICSI with mature oocytes and fresh sperm were done.

Luteal phase support started on the day of oocyte retrieval. All patients received vaginal micronized progesterone in gel form (Crinone 8%; Merck Serono, Istanbul, Turkey) in a single daily administration. Progesterone was used until ten weeks if pregnancy was confirmed.

Endometrial thickness determination: A transvaginal ultrasound scan was adopted on the day of hCG injection. The maximum distance between the two outer edges of the endometrial image on a longitudinal section of the uterus observed using a vaginal B-ultrasound (Voluson 730 Expert; General Electric Healthcare, Solingen, Germany) was used to determine endometrial thickness. Measurement of endometrial thickness in the pretreatment cycle was performed in the period following ovulation. In patients without ovulation, the highest endometrial thickness measurement was taken as a baseline for a week from the beginning of the midcycle. In patients in the ICSI cycle, the endometrial thickness measurement on hCG day was performed immediately before G-CSF infusion. No adverse reaction related to intrauterine G-CSF administration was reported in this study.

### Statistical analysis

All statistical analyses were performed using SPSS for Windows version 11.5 software (SPSS Inc., Chicago, IL, US). Descriptive statics were expressed as mean±standard deviation (SD) and median (min-max) for numerical variables, and in numerical form (percentage) for categorical variables. Student’s t-test was used to analyze statistically significant differences between the categories of a qualitative variable with two categories regarding a quantitative variable for normally distributed data, while a Mann-Whitney U-test was used for the abnormally distributed data. Chi-square and Fisher exact tests were used to analyze the relationship between two categorical variables. A *p*-value of 0.05 was considered statistically significant.

### Ethics approval and consent to participate

All procedures performed in studies involving human participants were in accordance with the ethical standards of the institutional and/or national research committee and with the 1964 Helsinki declaration and its later amendments or comparable ethical standards. (Ethics Code: 9 March 2016/99950669/75). The eligible patients signed written informed consent prior to participation in the study.

## Results

In total, 157 patients with RIF diagnoses were included in this study, with 82 patients (52.2%) in the G-CSF group and 75 patients (47.7%) in the control group. The demographic characteristics and pre-cycle identifiers of these individuals and their spouses are shown in Table 1.

**Table 1:** Demographic characteristics of patients in G-CSF and control groups, and a comparison of pre-cycle identifiers.

Variables	Groups				p value
	G-CSF (n = 82)		Control (n = 75)		
	Mean ± SD	Median (Min-Max)	Mean ± SD	Median (Min-Max)	
Female Age (year)	34.61 ± 4.77	35.00 (24.00–39.00)	34.92 ± 5.60	35.00 (22.00–39.00)	0.709^a^
Male Age (year)	37.79 ± 5.48	37.00 (26.00–59.00)	36.64 ± 5.76	35.00 (27.00–57.00)	0.226^a^
BMI (kg/m^2^)	25.92 ± 4.44	24.84 (18.34–39.56)	24.94 ± 4.92	24.09 (16.26–43.21)	0.197^a^
FSH (mIU/mL)	7.78 ± 3.97	6.25 (2.70–15.00)	8.93 ± 5.85	7.70 (3.12–15.00)	0.471^b^
AMH (ng/mL)	1.79 ± 1.54	1.30 (0.06–6.60)	1.92 ± 1.70	1.29 (0.01–6.60)	0.990^b^
AFC	8.99 ± 5.38	8.00 (2.00–20.00)	9.53 ± 5.82	8.00 (2.00–20.00)	0.658^b^
Duration of Infertility (year)	8.91 ± 4.86	8.00 (0.67–24.00)	6.69 ± 4.77	6.00 (0.42–20.00)	0.002^b^
Number of Previous Pregnancy	1.35 ± 1.82	1.00 (0.00–9.00)	1.16 ± 1.64	0.00 (0.00–7.00)	0.444^b^
Number of previous parity	0.12 ± 0.37	0.00 (0.00–2.00)	0.19 ± 0.43	0.00 (0.00–2.00)	0.244^b^
Miscarriages	0.96 ± 1.48	0.00 (0.00–7.00)	0.85 ± 1.39	0.00 (0.00–7.00)	0.698^b^
Number of Living Children	0.10 ± 0.37	0.00 (0.00–2.00)	0.15 ± 0.36	0.00 (0.00–1.00)	0.152^b^
Number of Previous IVF Cycle	3.09 ± 0.29	3.00 (3.00–9.00)	3.04 ± 0.20	3.00 (3.00–8.00)	0.215^b^
Number of Previous Failed IVF Cycle	3.08 ± 0.27	3.00 (3.00–7.00)	3.03 ± 0.16	3.00 (3.00–8.00)	0.170^b^
Sperm Count (million/mL)	44.91 ± 25.19	43.00 (1.10–127.00)	41.44 ± 26.09	39.00 (0.40–113.00)	0.413^a^

^a^Student t-test.

^b^Mann-Whitney U-test.

Abb. BMI:Body mass index AMH:Antimullerian hormone AFC:Antral follicle count IVF:Invitro fertilization.

In the G-CSF group, 13 couples (15.8%) were secondary infertile, and 69 couples (84.1%) were primary infertile, while in the control group, 14 couples (18.6%) were secondary infertile and 61 couples (81.3%) were primary infertile. No statistically significant difference was identified between the G-CSF and control groups for the primary and secondary infertility rates (p = 0.612).

No statistically significant difference was identified between groups for the presence/absence of Polycystic Ovary Syndrome (PCOS) (p = 0.851). The numbers (percentage) of PCOS presence and absence in the G-CSF group was found to be 8 (9.8) and 74 (90.2), respectively, compared to 8 (10.7) and 67 (89.3) respectively in the control group.

No statistically significant difference was identified between the two groups regarding the presence/absence of the male factor (p = 0.301). The numbers (percentage) of male factor presence and absence in the G-CSF group was found to be 17 (20.7) and 65 (79.7), respectively, compared to 19 (25.3) and 56 (74.6) respectively in the control group. Testicular sperm extraction procedure (TESE) was performed when no sperm was found in the ejaculate of two patients (2.5%) in the G-CSF group and three patients (4%) in the control group, and no statistically significant difference was identified between the two groups regarding the number of TESE applied (p = 0.672).

The characteristics of the ICSI cycle of our study are given in Table 2, with a comparison of the G-CSF and control groups. No statistically significant difference was identified between the two groups regarding controlled ovarian hyperstimulation protocols (p = 0.658). The numbers and percentages of antagonist and long-agonist protocol use were found to be 64 (78.0) in the G-CSF group and 18 (21.9) compared to 56 (74.6) and 19 (24.0) in the control group. There was no statistically significant difference between the groups regarding urinary and recombinant hCG use (p = 0.260). The numbers (percentages) of the urinary hCG and recombinant hCG categories were found to be 3 (3.6) and 79 (96.3) in the G-CSF group, compared to 4 (4.8) and 71 (94.6) in the control group.

**Table 2:** Comparison of ICSI cycle-related parameters between G-CSF and control groups.

Variables	Groups				p value
	G-CSF (n = 82)		Control (n = 75)		
	Mean ± SD	Median (Min-Max)	Mean ± SD	Median (Min-Max)	
Duration of Gonadotropin (day)	8.13 ± 1.61	8.00 (6.00–12.00)	8.16 ± 1.49	8.00 (5.00–14.00)	0.733^b^
Gonadotropin Dose (UI)	2295.00 ± 762.65	2137.50 (900.00–4500.00)	2279.29 ± 621.83	2250.00 (750.00–5175.00)	0.493^b^
Number of Retrieved Oocytes	7.29 ± 4.62	6.00 (1.00–21.00)	7.58 ± 5.38	6.00 (1.00–30.00)	0.890^b^
Number of M2 (mature) Oocytes	5.59 ± 3.98	5.00 (1.00–16.00)	5.60 ± 4.45	4.50 (1.00–29.00)	0.893^b^
Number of Fertilized Oocyte	4.67 ± 3.67	3.00 (1.00–16.00)	4.22 ± 3.10	3.00 (1.00–14.00)	0.754^b^
Fertilization Ratio	0.84 ± 0.20	1.00 (0.25–1.13)	0.78 ± 0.23	0.77 (0.20–1.13)	0.077^b^
Good Quality Embryo	2.38 ± 2.13	2.00 (0.00–10.00)	2.01 ± 2.03	2.00 (0.00–11.00)	0.230^b^
Number of Embryos Transferred	1.97 ± 0.51	2.00 (1.00–2.00)	1.99 ± 0.47	2.00 (1.00–2.00)	0.903^b^
ET day	17.53 ± 5.81	21.00 (3.00–26.00)	16.32 ± 5.99	18.50 (2.00–25.00)	0.157^b^
Endometrial Thickness in the Pretreatment Midcycle (mm)	8.02 ± 1.94	8.50 (7.00–14.80)	8.68 ± 2.07	8.50 (7.00–14.70)	0.620^b^
Endometrial Thickness on HCG Day (mm)	8.92 ± 1.91	9.00 (7.20–15.90)	8.98 ± 1.81	9.00 (7.50–15.00)	0.965^b^
Endometrial Thickness on ET Day (mm)	9.44 ± 2.06	9.00 (7.70–14.40)	9.45 ± 1.78	9.05 (7.50–14.40)	0.784^b^

^a^Student t-test.

^b^Mann-Whitney U-test.

ET:Embryo transfer HCG:Human chorionic gonadotrophine.

In our study, a total of 290 embryos were transferred, including 152 in the G-CSF group and 138 in the control group. In the G-CSF group, 120 (78.9%) cleavage and 32 (21.1%) blastocyst embryo transfers were performed, whereas in the control group, 107 (77.5%) cleavage and 31 (22.5%) blastocyst embryos were transferred. No difference was observed between two groups regarding the cleavage and blastocyst embryo transfer rates (p = 0.809).

Table 3 compares the pregnancy outcomes of the two groups to whom G-CSF was administered and not administered, with five and two twin pregnancies observed in the G-CSF group and the control group, respectively, these pregnancies resulted in miscarriage(fetal loss before 20^th^ weeks of gestation) and early premature birth(birth between 20–28 weeks of gestation). No difference was identified regarding beta hCG positivity, which was considered as per cycle and per embryo transfer, clinical pregnancy and live birth rates between the groups. Chemical pregnancies, miscarriage, early premature birth and term birth rates of pregnancy were also similar between the two groups.

**Table 3:** Comparison of ICSI cycle outcomes and pregnancy outcomes in G-CSF administered and non-administered groups.

Variables	Groups				p value
	G-CSF		Control		
	n	%	n	%	
B-hCG Positivity per Cycle	31	37.8	25	33.3	0.559^a^
B-hCG Positivity per ET	31	20.4	25	18.1	0.623^a^
Clinical Pregnancy Rate Per Cycle	31	37.8	20	26.7	0.137^a^
Clinical Pregnancy Rate Per ET	31	20.4	20	14.5	0.187^a^
Live Birth Rate per Cycle	12	14.8	13	17.3	0.668^a^
Live Birth Rate per ET	12	7.9	13	9.4	0.644^a^
Chemical Pregnancy	4	4.9	6	8.0	0.521^b^
Miscarriage (Fetal loss before 20^th^ weeks)	7	8.5	2	2.7	0.171^b^
Early Premature Birth (Birth between 20–28 weeks)	5	6.1	1	1.3	0.213^b^
Live Birth	12	14.8	13	17.3	0.668^a^

^a^Chi-square test.

^b^Fisher exact test.

## Discussion

This prospective randomized controlled trial was designed to determine whether or not intrauterine G-CSF administration before ART in the RIF group had an effect on clinical pregnancy and live birth rates. No statistically significant difference was identified between the G-CSF group and the control group regarding clinical pregnancy rates, miscarriage rates and live birth rates, and no difference was observed between the two groups regarding endometrial thickness.

G-CSF in ART was first used by Gleicher on four treatment-resistant cases with thin endometrium, and pregnancy was reported in all four cases^11^. In a single pilot cohort study carried out by Gleicher *et al*.^12^ in 2013, expansion was reported to be developed in 21 cases with thin endometrium that was resistant to standard therapies, and ongoing pregnancy was reported at a rate of 19.1%. In recent years, pregnancy rates have been reported to increase in the majority of trials in which intrauterine G-CSF was used in thin endometrium cases^15,18–20^. A study by Li *et al*.^21^, intrauterine G-CSF administration in the thin endometrium in frozen embryo transfer cycles were analyzed, and it was reported that this did not increase implantation or clinic pregnancy rates. Different from other studies, Eftekhar and Singh reported increased pregnancy rates, although no endometrial thickness increase was shown. In a meta-analysis by Kamath *et al*., this situation was reported as follows: “The higher pregnancy rates in spite of no apparent benefit in increasing endometrial thickness following G-CSF installation suggests the possibility of an unknown immunological mechanism aiding embryo implantation rather than only endometrial regeneration in both these populations”^7^. In our study, patients with thin endometrium were excluded, since the embryos were frozen in the event of endometrium at a thickness of less than 7 mm. In the present study, no difference was observed between the groups regarding endometrial thickness in the pretreatment cycle, and no difference was identified between the groups regarding endometrial thicknesses on both the hCG day and the embryo transfer days in the ART cycle. In our study, endometrial thickness did not increase in the group for whom G-CSF was administered compared to the group who did not receive G-CSF. In a study carried out by Barad *et al*.^16^ in 2014, the effects of intrauterine G-CSF infusion on a non-selected IVF group with normal endometrial thickness were investigated, and endometrial thickness and IVF outcome were reported to be unaffected by intrauterine G-CSF infusion, which was similar to the findings of the present study.

In the literature, G-CSF was administered systemically through the subcutaneous route in studies where the G-CSF was administered in the RIF group, and an increase in pregnancy rates was reported in all studies^17,22,23^. In our study, G-CSF administration was performed via endometrial installation in the RIF group, although no difference was seen in the implantation and pregnancy rates between the G-CSF and non-G-CSF groups. When administered systematically, G-CSF was reported to have positive effects on oocyte maturation and embryonic development^9^, while in locally endometrial cavity applications, pregnancy rates may not have changed since the oocyte and embryo were deprived of this positive support. Since thin endometrium was excluded due to the nature of our study, it can be concluded that G-CSF was not beneficial in increasing pregnancy rates in normal endometrium. In a study carried out by Eftekhar *et al*.^24^ in 2016, that the findings showed that the intrauterine infusion of G-CSF did not affect the success of ART in normal endometrium cases.

Literature contains a limited number of studies on the use of G-CSF in ART, and the quality and the level of evidence of the studies that have been made are not very high, including considerable heterogeneity in the selection of the patient population, the means of administration of G-CSF, the dose of G-CSF, application times, and in the statistical interpretations of the study results. Furthermore, in the published studies, the optimal dose, duration of application and route of administration were not identified^7,25,26^. The systemic and local use of G-CSF may have different effects, and different mechanisms of action may come into play in different clinical situations. Thus, it would seem plausible that it should be considered separately. Although degradation of endometrial receptors is a common point in all of them, RIF, recurrent pregnancy loss and thin endometrium are very different from each other and are multifactorial entities, which leads us to believe that specific groups should be focused upon in these studies.

The present study can be considered to be the first study that investigated the effects of intrauterine G-CSF administration on clinical pregnancy and live birth rates in the RIF group, and designed as a randomized controlled study; it involves well-chosen study and control groups with similar characteristics. These factors stand as the most powerful aspects of our study. Placebo administration was performed for the control group simultaneously with the study group to ensure that the endometrial injury effect (depending on the catheter and instilled fluid) was equal in both groups. The most important limitation of our study our inability to create subgroups due to the limited number of patients. In a larger study, subgroups, such as a thin endometrium group, a systemic G-CSF administered group, and different G-CSF dose administered groups could be applied to make the study much more valuable. Future studies also need to be conducted to ascertain what the most effective dose of G-CSF is when administered by different routes. Additionally; in this study, PGT-A was not performed to the embryos, this is another important limitation of our study. In a study regarding RIF the use of PGT-A would be better in order to avoid the chance that aneuploid embryos are transferred back in uterus.Our study would have been more valuable if we had performed PGT-A to the embryos.

In conclusion, intrauterine G-CSF administration in RIF patients with normal endometrium did not alter the endometrial thickness, clinical pregnancy rates, or live birth rates. Further studies are needed to investigate the effect of G-CSF on endometrium and implantation rates in RIF.

## Data Availability

All datasets used and analyzed in this study are available from the corresponding author on request.
